# Interleukin-1β-induced matrix metalloproteinase-3 via ERK1/2 pathway to promote mesenchymal stem cell migration

**DOI:** 10.1371/journal.pone.0252163

**Published:** 2021-05-21

**Authors:** Chun-Hao Chang, Yun-Li Lin, Yeu-Sheng Tyan, Yun-Hsuan Chiu, Ya-Han Liang, Chie-Pein Chen, Jiahn-Chun Wu, Hwai-Shi Wang

**Affiliations:** 1 Institute of Anatomy and Cell Biology, School of Medicine, National Yang Ming Chiao Tung University, Taipei, Taiwan, ROC; 2 Department of Medical Imaging, Chung Shan Medical University Hospital, Taichung, Taiwan, ROC; 3 Division of High Risk Pregnancy, Mackay Memorial Hospital, Taipei, Taiwan, ROC; Università degli Studi della Campania, ITALY

## Abstract

Human umbilical cord Wharton’s jelly derived mesenchymal stem cells (hUCMSCs), a source of cell therapy, have received a great deal of attention due to their homing or migrating ability in response to signals emanating from damaged sites. It has been found that IL-1β possesses the ability to induce the expression of matrix metalloproteinase-3 (MMP-3) in bone marrow MSCs. MMP-3 is involved in cell migration in various types of cells, including glioblastoma, vascular smooth muscle, and adult neural progenitor cells. In this study, we proposed that IL-1β influences hUCMSCs migration involving MMP-3. The expression level of MMP-3 in IL-1β-induced hUCMSCs was verified using cDNA microarray analysis, quantitative real-time PCR, ELISA and Western blot. Wound-healing and trans-well assay were used to investigate the cell migration and invasion ability of IL-1β-treated hUCMSCs. In addition, we pre-treated hUCMSCs with interleukin-1 receptor antagonist, MMP-3 inhibitors (ALX-260-165, UK 356618), or transfected with MMP-3 siRNA to confirm the role of MMP3 in IL-1β-induced cell migration. Our results showed that IL-1β induced MMP-3 expression is related to the migration of hUCMSCs. Moreover, extracellular signal-regulated protein kinases 1 and 2 **(**ERK1/2) inhibitor U0126, p38 inhibitor SB205380, JNK inhibitor SP600125 and Akt inhibitor GSK 690693 decreased IL-1β-induced MMP-3 mRNA and protein expression. The migration and invasion ability analyses showed that these inhibitors attenuated the IL-1β-induced migration and invasion ability of hUCMSCs. In conclusion, we have found that IL-1β induces the expression of MMP-3 through ERK1/2, JNK, p38 MAPK and Akt signaling pathways to enhance the migration of hUCMSCs. These results provide further understanding of the mechanisms in IL-1β-induced hUCMSCs migration to injury sites.

## Introduction

Human umbilical cord Wharton’s jelly derived mesenchymal stem cells (hUCMSCs) are rapidly gaining attention for its therapeutic potential in regenerative medicine [1]. Stem cells migration toward the damaged tissues play critical roles in wound healing and tissue regeneration [2]. It has been found that damaged tissue may release factors that mobilize and recruit stem cells toward sites of injury, and then promote their proliferation and differentiation and eventually repair the damaged tissues [3, 4]. The local or systemic inflammatory state might influence not only MSC mobilization, but also therapeutic effects [5]. However, the mechanisms that guide MSCs to appropriate damaged microenvironments are not yet understood.

Numerous studies have shown that various cytokines or growth factors exert important effects with regard to inflammatory and ischemic tissue. Expression of stromal cell-derived factor 1 (SDF-1) has been detected in MSCs, which promote the homing ability of MSCs toward the ischemia-induced deteriorated heart muscle tissue [6]. Transforming growth factor (TGF)-β1, monocyte chemotactic protein (MCP)-1, tumor necrosis factor (TNF)-α, and interleukins (IL) have also been found to promote MSCs migration to the injured region [2, 7]. In addition, studies indicate that the migration capacity of MSCs is partly under control of a large range of receptor of cytokines and growth factors, including interleukin-1 receptor (IL-1R) or PDGF-receptor (R) [5, 8].

Interleukin-1β is an important mediator involved in the inflammatory response and tissue damage in various organs. It has been found that IL-1β induces mesenchymal stem cells migration and leukocytes chemotaxis [9]. Some studies indicated that IL-1β induced different types of matrix metalloproteinase (MMP) expression is association with cell migration [10–14].

Specific interest in MMP-3 (stromelysin-1) as a target has grown because of its broad tissue distribution and substrate specificity. MMP-3 breaks down collagen types III, IV, and V, fibronectin, elastin, proteoglycans and laminin, and is secreted by various kinds cell types including vascular smooth muscle cells (VSMCs), endothelial cells (ECs), and bone marrow MSCs [15, 16]. In particular, MMP-3 has been reported that is involved in the cellular migration in glioblastoma cells, VSMCs, and adult neural progenitor cells [17].

Previous studies indicated that inflammatory cytokines, such as IL-1β, tumor necrosis factor (TNF)-α, and transforming growth factor (TGF)-β1 increase the production of MMPs in MSCs, which stimulate a great chemotactic migration of MSCs through the extracellular matrix [2, 18].

IL-1β-mediated MMP-3 expression can be found in bone marrow MSCs [9], trabecular meshwork [19], and chondrocytes [20] reported that breast adipose MSCs readily penetrate extracellular matrix components in part through up-regulation of MMP-3, and then promote the invasive ability of Human Caucasian breast epithelial cell line T4-2 cells and efficiently chemoattract ECs [21].

On the whole, there are no studies that discuss the impact of MMP-3 on IL-1β-induced MSCs migration. Therefore, in this research we attempt to investigate the role of MMP-3 in IL-1β-induced mesenchymal stem cell migration and the signaling pathway of IL-1β-induced MMP-3 expression in MSCs.

## Materials and methods

### Cell culture

Human umbilical cord derived mesenchymal stem cells (hUCMSCs) were purchased from Bioresource Collection and Research Center, Hsinchu, Taiwan. hUCMSCs were cultured in low serum defined medium consisting of 56% low-glucose Dulbecco’s Modified Eagle Medium (DMEM-LG; Invitrogen, CA, USA), 37% MCBD 201 (Sigma, MO, USA), 2% fetal bovine serum (Thermo, Logan, UT), 0.5 mg/ml of AlbuMAX® I (Invitrogen, CA, USA), 1X insulin-transferrin-selenium-A (Invitrogen, CA, USA), 1X antibiotic antimycotic solution (Thermo, Logan, UT), 10 nM dexamethasone (Sigma, MO, USA), 50 nM L-ascorbic acid 2-phosphate (Sigma, MO, USA), 10 ng/ml of epidermal growth factor (PeproTech, NJ, USA), and 1 ng/ml of platelet-derived growth factor-BB (PeproTech, NJ, USA) at 37°C and 5% CO_2_. When cells reached 70–80% confluence, the cells were detached by using HyQtase (Thermo, Logan, UT) and replated at a ratio of 1:4.

### Cytokines and inhibitors

hUCMSCs were starved in serum-free DMEM-LG containing 0.1% bovine serum albumin (BSA) for 16 hours, then treated with 2 μg/ml IL-1 receptor antagonist (IL-1RA) (Peprotech, NJ, USA), MMP-3 inhibitors UK356618 (20 nM) (Tocris, UK) and ALX260165 (20 μM) (Enzo life, UK), ERK1/2 inhibitor U0126 (10–30 μM) (Tocris, UK), p38 inhibitor SB205380 (50 nM) (Tocris, UK), Akt inhibitor GSK 690693 (20 μM) (Tocris, UK) and JNK inhibitor SP600125 (20 nM) (Tocris, UK) for 2 hours prior to human recombinant interleukin-1β (IL-1β) stimulation. Cells were then incubated with 100 ng/ml IL-1β (Peprotech, NJ, USA) in the continued presence of these inhibitors for 12–48 hours.

### Cell viability assay

hUCMSCs were seeded in 96-well plates in serum-free DMEM-LG containing 0.1% BSA for 16 hours and then stimulated with 100 ng/ml IL-1β for 36 hours. MTT assay reagent (3-(4,5-Dimethyl-2-thiazolyl)-2,5-diphenyl-2H-tetrazolium bromide, SERVA Heidelberg German 20395) were added into the culture medium in 1 mg/mL and incubated with cells for 4 hours at 37°C and 5% CO2. Afterwards, the reagent is removed and MTT formazan is dissolved with DMSO (Sigma, MO, USA) for 2 hours. The MTT assay data were determined by Multimode microplate readers (Infinite 200, TECAN).

### Flow cytometry

hUCMSCs were starved in serum-free DMEM-LG medium containing 0.1% BSA for 16 hours and stimulated with or without 100 ng/ml IL-1β for 36 hours. Cells were washed twice with PBS and detached by HyQtase (Thermo, Logan, UT). Cell were then suspended 1*10^6^ cells in 100 μl PBS and incubated with 1:20 dilution of conjugated antibodies CD105-FITC (R&D systems, USA), CD34-FITC (Becton, Dickinson and Company, USA), CD73-PE (Becton, Dickinson and Company, USA), CD45-PE (Thermo Fisher Scientific, USA), CD90-PE (Beckman Coulter, USA) at 4°C for 30minutes on the shaker. The unstained cells were cultured with PBS only. Cells were then washed twice with PBS and centrifuged at 0.3 g for 5 minutes. Finally, cells were re-suspended in 200 μl PBS and immediately analyzed using Beckman Coulter CytoFLEX (Beckman, IL, USA).

### Microarray analysis

hUCMSCs were starved in serum-free DMEM-LG containing 0.1% BSA for 16 hours and stimulated with or without 100 ng/ml IL-1β for 24 hours. Total RNA purification was performed using TriPure isolation reagent (Bioline, London, UK) according to the manufacturer’s instructions. RNA qualities were checked by RNA electrophoresis before hybridizing with GeneChip™ Human Genome U133 Plus 2.0 Array (Affymetrix array, Thermo Fisher Science, CA, USA). The cDNA detection and raw data were analyzed by National Yang-Ming University VYM Genome Research Center.

### Quantitative real-time polymerase chain reaction

The total RNA extraction from hUCMSCs with or without IL-1β stimulation was extracted by using TriPure isolation reagent (Bioline, London, UK) according to the manufacturer’s instruction. RNA was converted to cDNA with the Tetro cDNA Synthesis kit (Bioline, London, UK). The following oligonucleotides were used for each gene:

MMP-3 forward primer 5’-TGGACAAAGGATACAACAGGGAC-3’

MMP-3 reverse primer 5’-AGCTTCAGTGTTGGCTGAGT-3’

GAPDH forward primer 5’-GAAGGTGAAGGTCGGAGTCAAC-3’

GAPDH reverse primer 5’-CAGAGTTAAAAGCAGCCCTGGT-3’

Gene expression was analyzed by quantitative real-time PCR using SensiFAST CYBR Hi-ROX System (Bioline, London, UK) and each reaction was repeated in triplet.

### MMP-3 enzyme-linked immunosorbent assay

The condition medium of IL-1β stimulated hUCMSCs for 36 hours was collected to quantitate MMP-3 protein expression and activity using MMP-3 Human ELISA Kit (Lifetechnologies (Novex), USA). The results were detected using a Spectrophotometer reader (ND-1000, NanoDrop) at a wavelength of 450 nm.

### Western blotting

To prepare the cell lysates for Western blot, hUCMSCs were washed with PBS and lysed using M-PER mammalian protein extraction reagent (Thermo, IL, USA) with Halt protease inhibitor cocktail (Thermo, IL, USA), then placed in a centrifuge at 14,000 g for 10 min at 4°C to collect the precleared cell extracts. Protein concentration of the samples were determined using the Coomassie Plus (Bradford) protein assay reagent (Thermo, IL, USA) and Multimode microplate readers (Infinite 200, TECAN). Protein samples were separated using 10% sodium dodecyl sulfate-polyacrylamide gel electrophoresis and then transferred to polyvinylidene fluoride (PVDF) membranes (Merck, Darmstadt, Germany). Membrane blocking solution 10% Fish gelatin blocking buffer (AMRESCO, OH, USA) was used to block PVDF membranes for 1 hour. Then membranes were incubated with the anti-human MMP-3 primary antibody (R&D systems, USA) at 1:1000 dilution at 4°C overnight, followed by washing with tris-buffered saline with tween 20 (TBST). The membranes were then incubated with goat anti-mouse secondary antibody at room temperature for an hour. The western blot data were detected by enhanced chemiluminescence substrate using Luminescence Imaging System (LAS-4000, GE, USA).

### Fluorogenic assays of protease activity of MMP-3

After stimulation with IL-1β for 36 hours, the condition medium was collected. Quantitation of MMP-3 activity in the conditioned medium was analyzed by using Fluorogenic peptide substrate II (R&D systems, USA). The protease activity of MMP-3 was detected at emission and excitation wavelengths of 320 nm and 405 nm, respectively, in kinetic mode for 5 min using a Multimode microplate reader (Infinite 200, TECAN).

### Wound healing assay

Equal number of hUCMSCs were inoculated into cell culture insert (Ibidi, Planegg, Germany) in 12-well plates to grow until confluence. Prior to the addition of inhibitors, hUCMSCs were starved in serum-free DMEM-LG containing 0.1% BSA for 16 hours. Then cells were incubated with IL-1RA (2μg/ml), UK356618 (20 nM), ALX260165 (20 μM) or U0126 (20 μM) for 2 hours before treatment with or without 100 ng/ml IL-1β (100 ng/ml) for 12–24 hours. Migrated cells were observed with an inverted microscope and photographs were taken in the same field every 12 hours after stimulation for 24 hours.

### *In vitro* invasion assay

The *in vitro* invasion assay was performed in an 8.0-μm pore size matrigel invasion chamber (CORNING, MA, USA). hUCMSCs were seeded into 6-well plates and grew until confluence. Cells were starved in serum-free DMEM-LG containing 0.1% bovine serum albumin for 16 hours, then incubated with IL-RA (2 μg/ml), UK356618 (20 nM), ALX260165 (20 μM) or ERK1/2 inhibitor U0126 (20 μM) for 2 hours. Afterwards, hUCMSCs were incubated for 36 hours with 100 ng/ml IL-1β (Peprotech, NJ, USA) in the continued presence of these inhibitors. Cells were detached with HyQtase (Thermo, Logan, UT), and 1.5×10 ^4^ cells were plated into the upper matrigel chamber in serum-free DMEM-LG with hUCMSCs growth medium added to the lower chamber. After 24 hours of incubation at 37°C and 5% CO_2_, non-migrated cells in the upper chamber were scraped with a cotton swab. Cells that had migrated to the lower chamber were fixed and stained with crystal violet (Sigma, MO, USA). Microscopy was used to count the migrated cell numbers.

### siRNA transfection

MMP3 Silencer Select Pre-designed siRNA (s8854 and s8855, Ambion, Austin, USA) and Silencer Select negative control #1 (Ambion, Austin, USA) were used to downregulate MMP3 expression in cells. Cells were plated in 6 well plates 24 hours prior to transfection with 5 nM of MMP3 specific siRNA or negative control siRNA using Lipofectamine RNAiMAX Transfection Reagent (Invitrogen, CA, USA).

### Statistical analysis

Statistical analyses were performed using SPSS software (version 16.0). Quantitation data were analyzed by Student’s t-test and one-way ANOVA. P values <0.05 were considered statistically significant.

## Results

### IL-1β stimulates mesenchymal stem cell migration

**Fig 1:**
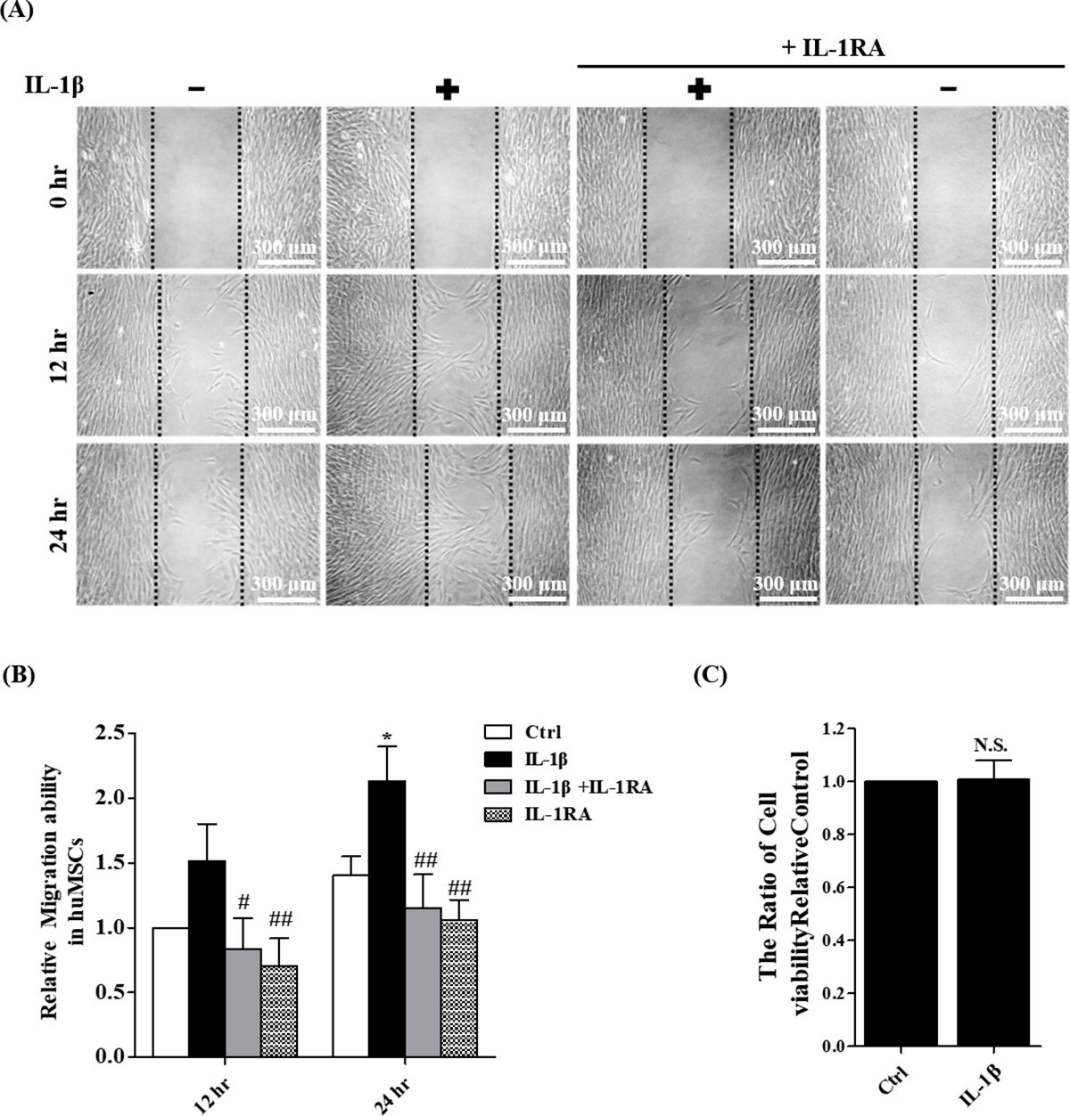
IL-1β stimulates mesenchymal stem cell migration. (A) Cell wound healing assay for IL-1β stimulated mesenchymal stem cells in the presence or absence of 2 μg/ml IL-1RA (IL-1β inhibitor) at 12 and 24 hours. Scale bars = 300 μm. (B) The wound area of hUCMSCs were indicated by MetaMorph and the data were normalized with control and shown as the mean ± SD (n = 3, *P<0.05, **P<0.01 versus control cells, #p<0.05, ##p<0.01 versus IL-1β treated cells). (C) Cell viability assay for IL-1β stimulation. Data were quantified by multimode micro-plate readers. Data are shown as the mean ± SD (n = 3) (N.S.: nonsignificance).

The ability of IL-1β to induce human MSC migration *in vitro* was examined by wound healing assay. In our previous study, we examined the cell viability of hUCMSCs treated with 0 to 500 ng/ml IL-1β. The result showed that treatment with IL-1*β* at a concentration of 100 ng/ml (the concentration we used in this manuscript) showed no significant change. MTT assay also showed no significant change in 100 ng/ml IL-1*β*-treated hUCMSCs in comparison to the control group [22]. In this study, 100 ng/ml of IL-1β was used to treat hUCMSCs for 24 hours, the stem cell migration was significantly enhanced. This effect was blocked by 2 h pretreatment with the IL-1β receptor antagonist IL-1RA (Fig 1A and 1B). The cell viability assay data showed no significant difference between IL-1β-treated and control groups (Fig 1C), suggested that IL-1β stimulated stem cell migration was not affected by cell viability.

### Effects of IL-1β in hUCMSCs on MMP-3 RNA and protein levels

**Fig 2:**
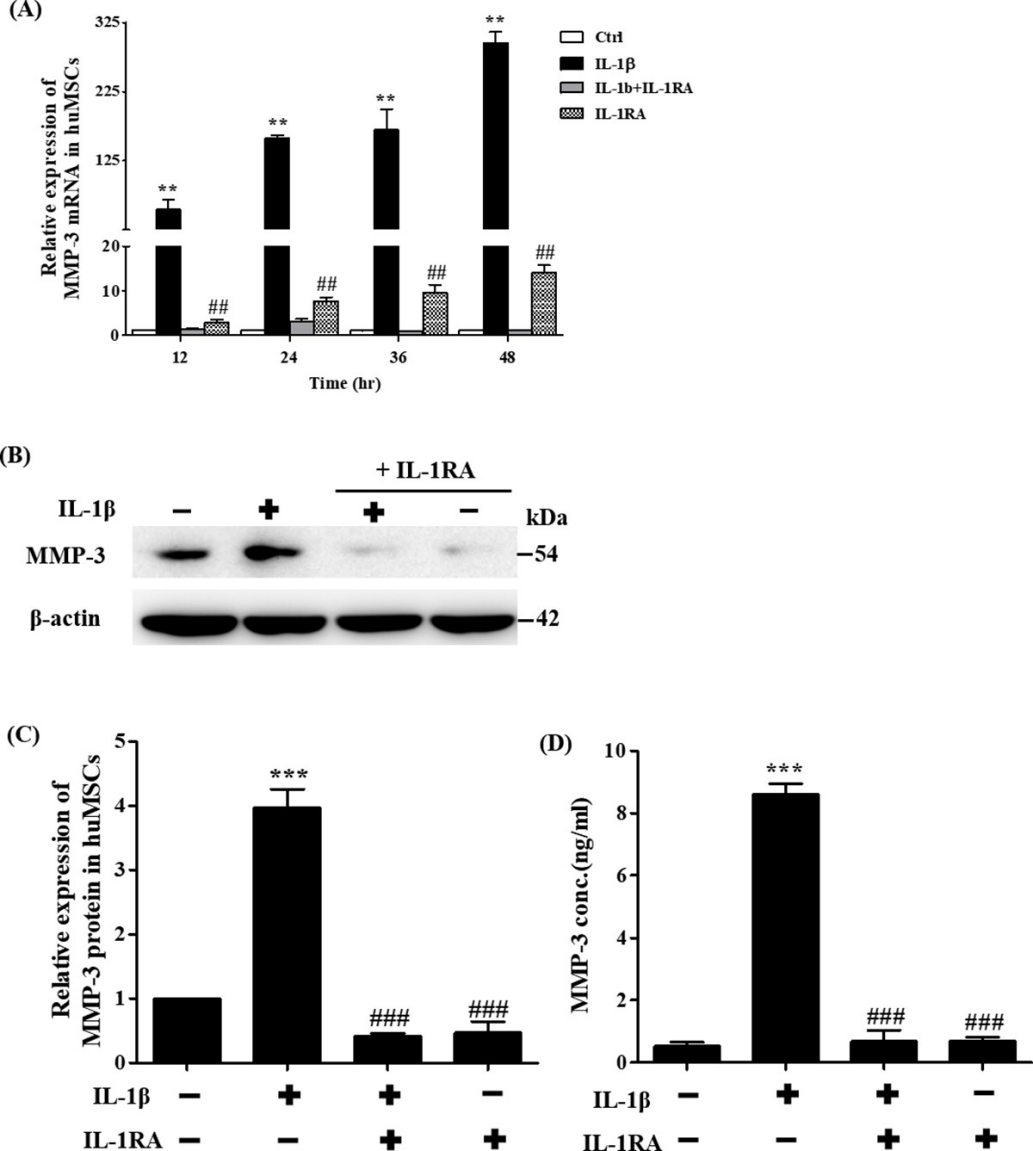
Effects of IL-1β in hUCMSCs on MMP-3 RNA and protein levels. (A) Quantitation of changes in gene expression of MMP-3 detected by real-time PCR after IL-1β inhibitor IL-1RA (2 μg/ml) pre-treatment and stimulation with IL-1β for 12–48 hours. (B) Example of Western blot results of the MMP-3 (54 kDa) from the lysates of cells treated with IL-1β and IL-1RA. The full-length Western blots was showed in S2 Fig. (C) Quantitative graphs of the Western blot results of MMP-3 protein expression of (B). (D) MMP-3 protein expression was measured using ELISA, pre-treatment with IL-1RA at concentration of 2 μg/ml and stimulated with IL-1β for 36 hours. Data are shown as the mean ± SD (n = 3, **P<0.01, ***P<0.005 versus control cells, #p<0.05, ##p<0.01 versus IL-1β treated cells).

To determine the molecular pathways that are involved in the migration of mesenchymal stem cells induced by IL-1β, gene expression profiles of IL-1β treated and untreated control cells were identified by cDNA microarray (Human Genome U133 2.0 Array, Affymetrix). The results showed that MMP-3 was up-regulated significantly in IL-1β treated mesenchymal stem cells (Table 1). The mRNA expression of MMP-3 was detected by quantitative real-time PCR, the data showed that the level of MMP-3 transcript in hUCMSCs that were treated with IL-1β for 12 hours were significantly higher than untreated cells (Fig 2A). These findings were consistent with our Human Genome U133 2.0 Array Chip data. To clarify that the MMP-3 expression was induced by IL-1β, cells were pretreated with IL-1β receptor antagonist IL-1RA, the results showed that IL-1RA significantly suppressed IL-1β-induced MMP-3 expression. To investigate whether IL-1β could induce MMP-3 protein expression in mesenchymal stem cells, MMP-3 Western blotting analysis was performed. The level of MMP-3 protein expression in IL-1β treated cells was higher than non-treated cells. IL-1β-induced MMP-3 protein expression can be suppressed by IL-1RA (Fig 2B and 2C). To further confirm the secretion level of MMP-3 of stem cells treated with IL-1β, the secretion level of MMP-3 protein expression in the cell medium was quantified using MMP-3 ELISA assay. As shown in Fig 2D, MMP-3 expression was significantly higher in IL-1β-treated stem cells compared with control. IL-1β induced MMP-3 expression was inhibited by IL-1RA (Fig 2D). Taken together, these results showed that IL-1β promotes MMP-3 protein expression and increases secretion level of MMP-3 in mesenchymal stem cells culture medium.

**Table 1:** Total RNA microarray based screening for the expression of MMPs with IL-1β stimulation for 24 hours in mesenchymal stem cells.

Gene Symbol	Gene Title	Log Ratio
MMP3	Matrix metallopeptidase 3 (stromelysin 1, progelatinase)	3.8
MMP19	Matrix metallopeptidase 3	1.6
MMP10	Matrix metallopeptidase 3 (stomelysin 2)	1.2

### Effects of MMP-3 expression on IL-1β-induced cell migration and invasion

**Fig 3:**
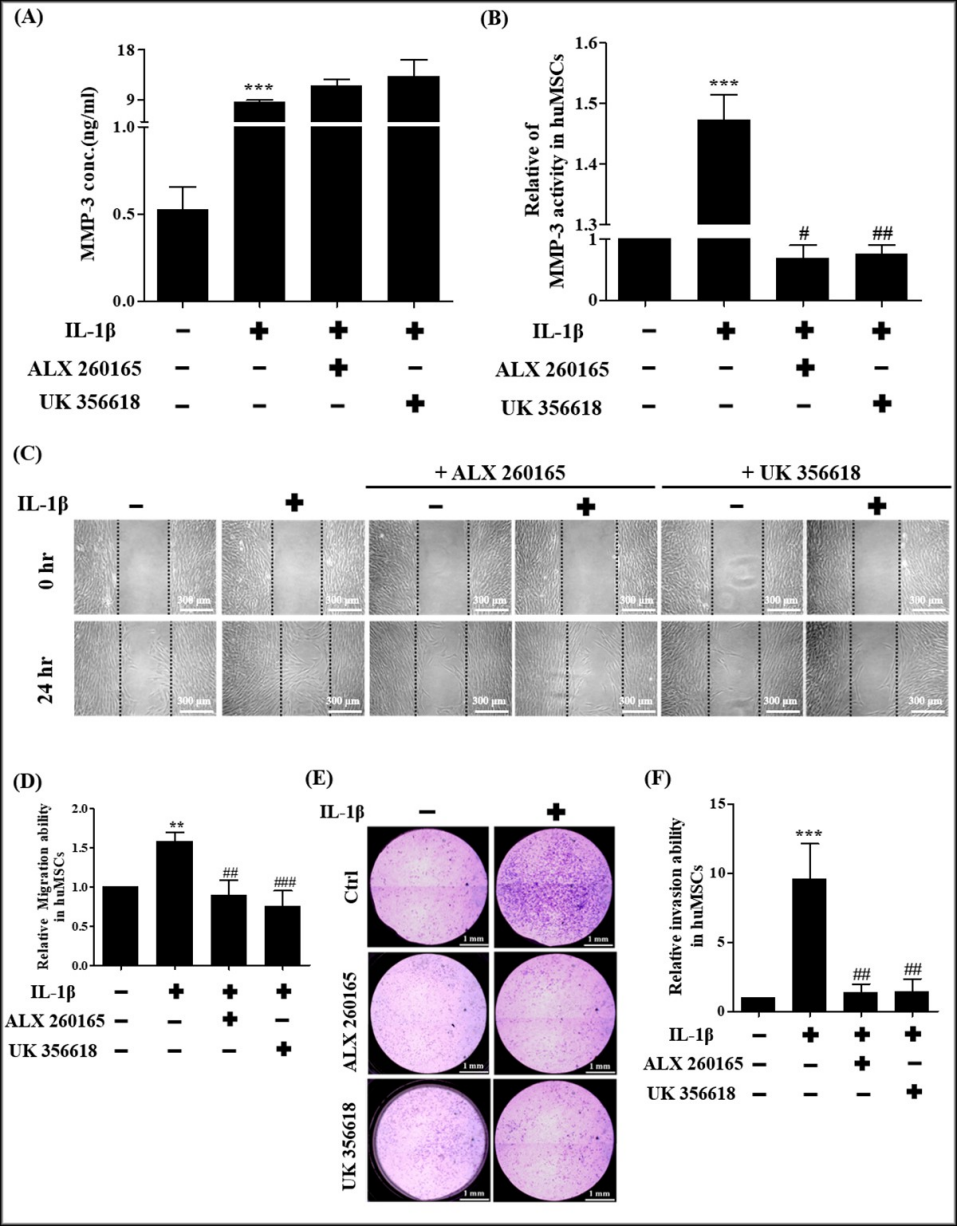
Effects of MMP-3 inhibitor in hUCMSCs on MMP-3 secretion and MMP3 activity, cell migration and invasion. (A) MMP-3 protein expression measured by ELISA. hUCMSCs treated with ALX 260165 and UK 356618 at concentration of 20 μM. (B) MMP-3 activity was measured by Fluorogenic peptide Assays. hUCMSCs treated with ALX 260165 and UK 356618 at concentration of 20 μM. (C) Cell wound healing assay. Cultures were treated with MMP-3 inhibitors ALX 260165, UK 356618 at concentrations of 20 μM, 20 nM, respectively as indicated. Scale bars = 300 μm. (D) Quantitative graph showing the migration ability of stem cells into the wound area at 24 hours. (E) Cell invasion assay, cultures were treated with MMP-3 inhibitors ALX 260165, UK 356618 at concentrations of 20 μM, 20 nM, respectively as indicated. Scale bars = 1 mm. (F) Graph indicates the invasion ability of stem cells. Data are shown as the mean ± SD (n = 3, **P<0.01, ***P<0.005, versus control cells, #p<0.05, ##p<0.01, ###p<0.005 versus IL-1β treated cells).

To determine whether IL-1β induced mesenchymal stem cell migration ability was influenced by the expression of MMP-3, MMP-3 inhibitors ALX 260165 and UK 356618 were used. The results revealed that stem cells treated with ALX 260165 and UK 356618 did not decrease the secretion level of MMP-3 (Fig 3A). By using fluorogenic peptide assay, the results showed that these inhibitors could reduce MMP-3 activity (Fig 3B). The migration ability was significantly higher in IL-1β-treated stem cells in comparison with untreated control after 24 hours, cells treated with ALX 260165 or UK 356618 can significantly attenuate IL-1β-induced cell migration (Fig 3C and 3D). This study demonstrated that IL-1β induced mesenchymal stem cell migration ability was influenced by MMP-3 expression and MMP-3 activity. To further investigate whether IL-1β-induced mesenchymal stem cell invasion ability was influenced by the expression of MMP-3, MMP-3 inhibitors ALX 260165 and UK 356618 were used in cell invasion assay. As shown in Fig 3E and 3F, the invasion ability was significantly higher in IL-1β-treated stem cells in comparison with untreated control cells after 36 hours. The result indicated that cells treated with ALX 260165, UK 356618 can significantly attenuate IL-1β-induced cell invasion (Fig 3E and 3F). This result demonstrated that IL-1β induced mesenchymal stem cell invasion ability was influenced by the MMP-3 expression and MMP-3 activity.

### Knockdown of MMP-3 expression suppresses IL-1β-induced cell migration and invasion

**Fig 4:**
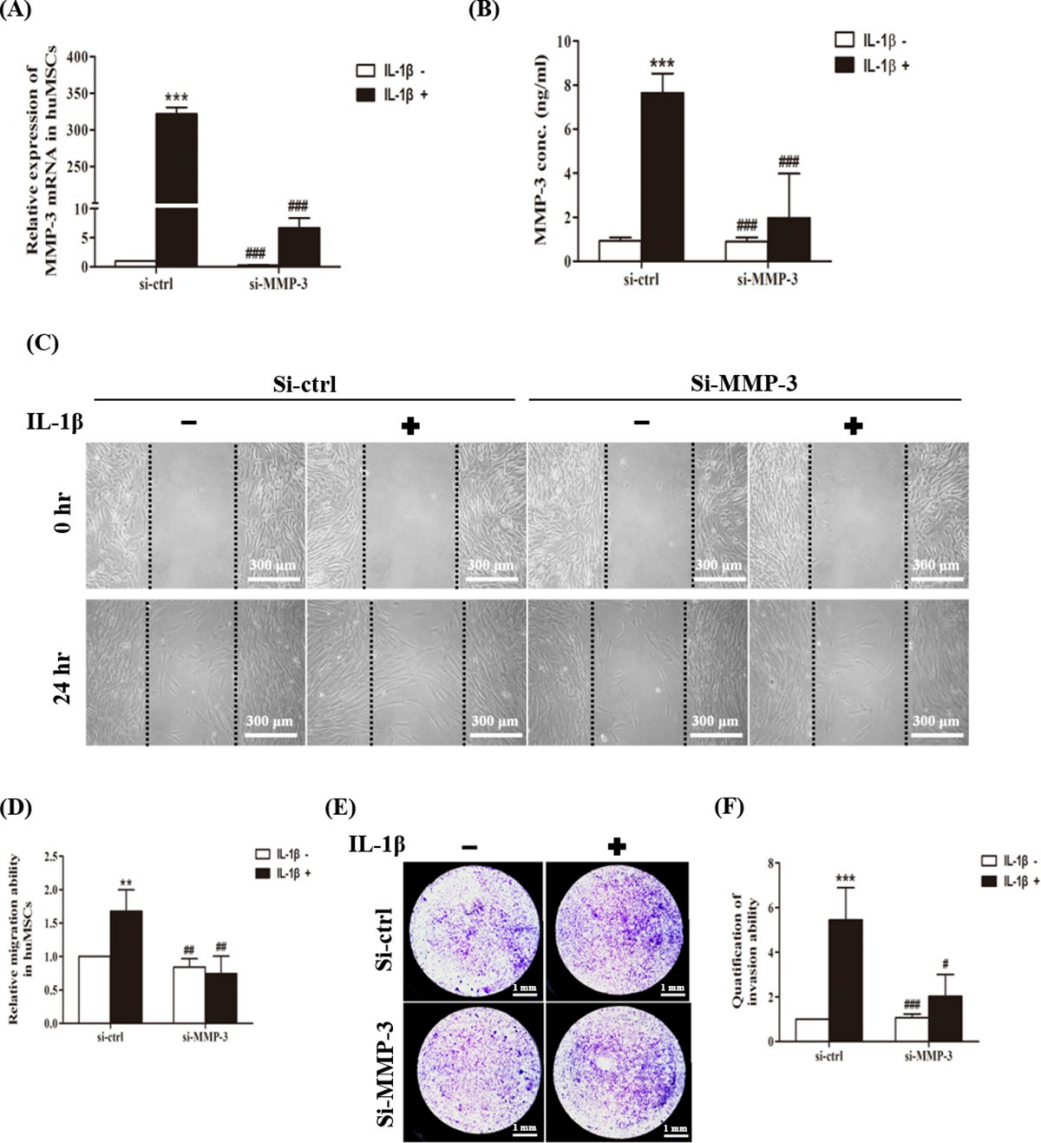
Effect of knockdown MMP-3 on IL-1β treated hUCMSCs. (A) Quantitation of changes in gene expression of MMP-3 detected by real-time PCR. hUCMSCs were transfected with MMP-3 siRNA and stimulated with IL-1β for 24 hours. (B) MMP-3 protein expression was measured using ELISA, transfected with MMP-3 siRNA and stimulated with IL-1β for 36 hours. (C) Cell wound healing assay for IL-1β stimulated mesenchymal stem cells after MMP-3 siRNA transfection. Scale bars = 300 μm. (D) The wound area of hUCMSCs were indicated by MetaMorph. (E) Cell invasion assay for IL-1β stimulated mesenchymal stem cells after MMP-3 siRNA transfection. Scale bars = 1 mm. (F) Graph indicates the invasion ability of stem cells. Data are shown as the mean ± SD (n = 3, **P<0.01, ***P<0.005 versus control cells, #p<0.05, ##p<0.01, ###p<0.005, versus IL-1β treated cells).

After establishing the signaling pathways that are involved in IL-1β-induced MMP-3 expression, we examined whether MMP-3 expression was required for hUCMSCs migration by MMP-3 siRNA. Real time PCR and ELISA data showed that siRNA-MMP-3 transfection of hUCMSCs resulted in knockdown of MMP-3 mRNA levels (Fig 4A) and protein levels (Fig 4B) and suppressed IL1-β-induced cell migration in wound healing assays (Fig 4C and 4D). The cell invasion assay showed that cell invasion ability of IL-1β-treated MMP-3-siRNA-transfected hUCMSCs was decreased in comparison with that of the IL-1β-treated control-siRNA-transfected hUCMSCs (Fig 4E and 4F). These results show that a reduction in MMP-3 expression by siRNA blocks the IL-1β-induced migration of hUCMSCs.

### Effects of ERK1/2 pathway on IL-1β-mediated MMP-3 mRNA, protein, MMP-3 activity and cell migration

**Fig 5:**
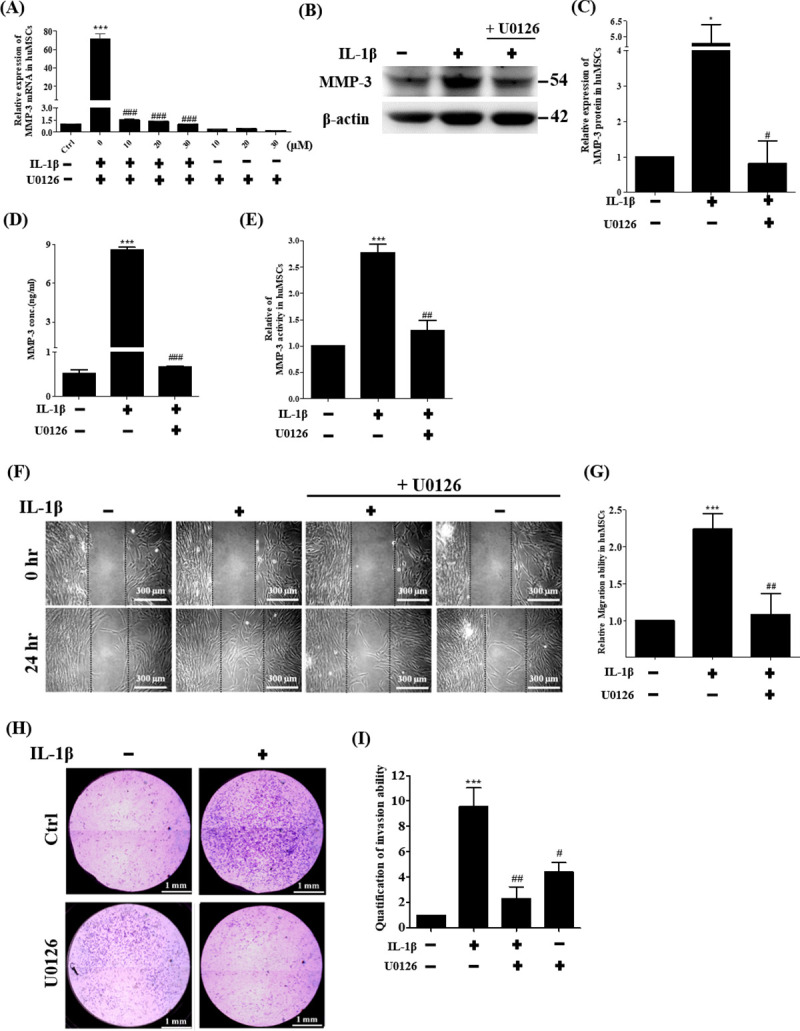
Effects of ERK1/2 pathway on IL-1β-mediated MMP-3 expression, cell migration and cell invasion. (A) Quantitation of changes in gene expression of MMP-3 detected by real-time PCR after ERK1/2 inhibitor U0126 (10–30 μM) treatment and stimulation with IL-1β for 24 hours. (B) Example of Western blot results of the MMP-3 (54 kDa) from the lysates of cells treated with IL-1β and ERK1/2 inhibitor U0126 (20 μM). The full-length Western blots was showed in S2 Fig. (C) Quantitative graphs of the Western blot results of MMP-3 protein expression of (B). (D) MMP-3 protein expression was measured using ELISA, treated with U0126 (20 μM) and stimulated with IL-1β for 36 hours. (E) MMP-3 activity was measured by Fluorogenic peptide Assays. hUCMSCs treated with U0126 at concentration of 20 μM. (F) Cell wound healing assay for IL-1β stimulated mesenchymal stem cells in the presence of ERK1/2 inhibitor U0126 (20 μM) at 24 hours. Scale bars = 300 μm. (G) The wound area of hUCMSCs were indicated by MetaMorph and the data were normalized with control and shown as the mean ± SD (n = 3, ***P<0.01 versus control cells, ##p<0.01 versus IL-1β treated cells). (H) Cell wound healing assay for IL-1β stimulated mesenchymal stem cells in the presence of ERK1/2 inhibitor U0126 (20 μM) at 24 hours. Scale bars = 1 mm. (I) Graph indicates the invasion ability of stem cells. Data are shown as the mean ± SD (n = 3, *P<0.05, *** P <0.005 versus control cells, #p<0.05, ##p<0.01, ###p<0.005 versus IL-1β treated cells).

It has been observed that IL-1β induced MMP-3 expression via ERK1/2 activation in chondrocytes [20]. To investigate whether ERK1/2 plays a role in IL-1β-induced MMP-3 RNA expression in mesenchymal stem cells, MMP-3 quantitative real-time PCR analysis was performed in cells pretreated with ERK1/2 inhibitor U0126. Fig 5A, shows that the level of MMP-3 transcript in IL-1β treated cells was significantly higher than non-treated cells after 24 hours. Cells pretreated with ERK1/2 inhibitor U0126 IL-1β-induced MMP-3 RNA levels were significantly suppressed. This result demonstrated that IL-1β-induced the expression of MMP-3 RNA in mesenchymal stem cells via the ERK1/2 pathway.

To determine whether IL-1β-induced MMP-3 protein expression in mesenchymal stem cells via ERK1/2, ERK1/2 inhibitor U0126 was used to treat hUCMSCs then cells were analyzed by Western blot. Fig 5B and 5C shows that the level of MMP-3 protein expression in IL-1β treated cells was significantly higher than non-treated cells after 36 hours. The cells treated with ERK1/2 inhibitor U0126 showed that IL-1β-induced MMP-3 protein expression was significantly suppressed. The secretion level of MMP-3 in the stem cell medium treated with IL-1β was quantified by ELISA assay. As shown in Fig 5D, MP-3 expression was significantly higher in IL-1β-treated stem cells in comparison with untreated control cells after 36 hours. IL-1β induced MMP-3 expression in hUCMSCs was inhibited by ERK1/2 inhibitor U0126 (Fig 5D). The activity of MMP-3 of stem cells treated with IL-1β in the cell medium was quantified by Fluorogenic peptide Assays. As shown in Fig 5E, MMP-3 activity was significantly higher in IL-1β-treated hUCMSCs compared with untreated control cells after 36 hours. IL-1β induced MMP-3 activity in hUCMSCs was inhibited by ERK1/2 inhibitor U0126 (Fig 5E). Taken together, these results showed that IL-1β induced MMP-3 expression occurs via ERK1/2 pathway in mesenchymal stem cells.

To determine the role of ERK1/2 signaling pathway in IL-1β-induced cell migration, wound healing assays were performed in cultures treated with ERK1/2 inhibitor U0126. As shown in Fig 5F and 5G, migration ability was significantly higher in IL-1β-stimulated hUCMSCs in comparison with the control group after 24 hours. The results showed that the effect on the migration of IL-1β-induced stem cells was attenuated when ERK1/2 inhibitor U0126 was added to the cells (Fig 5F and 5G). Cell invasion assays were performed in transwells coated with matrigel. As shown in Fig 5H and 5I, the invasion ability was significantly higher in IL-1β-treated hUCMSCs in comparison with untreated control cells after 36 hours. Cell invasion of IL-1β-induced stem cells was attenuated when ERK1/2 inhibitor U0126 was added to the cells (Fig 5H and 5I). These results demonstrate that IL-1β-induced MMP-3 via ERK1/2 is involved in hUCMSCs migration and hUCMSCs invasion.

### Effects of JNK, p38, and Akt pathway on IL-1β induced MMP-3 expression and migration of hUCMSCs

**Fig 6:**
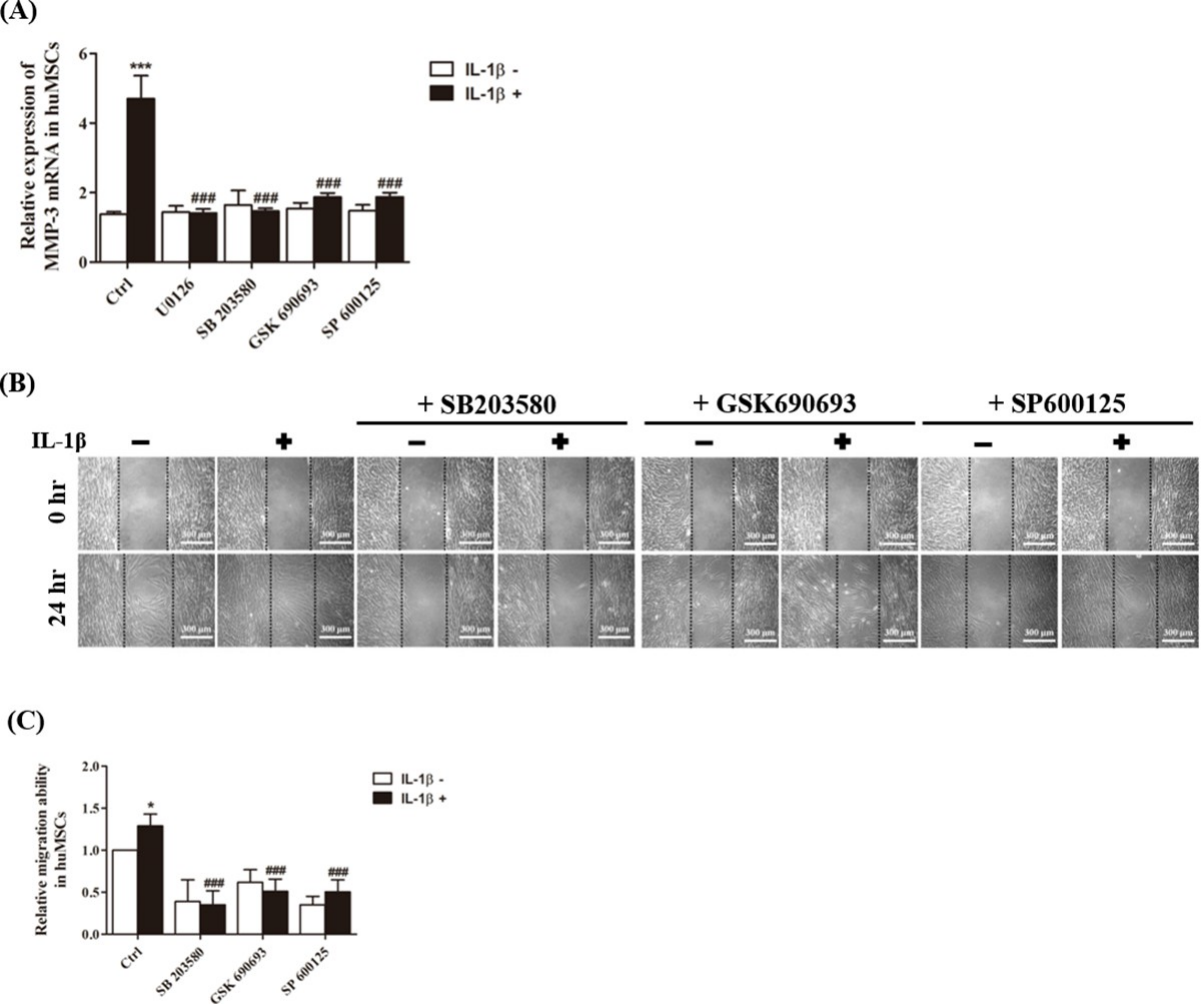
Effects of JNK, p38, and Akt pathways on IL-1β induced MMP-3 expression on hUCMSCs. (A) Quantitation of changes in gene expression of MMP-3 detected by real-time PCR after p38 inhibitor (50 nM SB205380), Akt inhibitor (20 μM GSK690693), and JNK inhibitor (20 nM SP600125) treatment and stimulation with IL-1β for 24 hours. (B) Cell wound healing assay for IL-1β stimulated mesenchymal stem cells in the presence 50 nM SB205380, 20 μM GSK690693, and 20 nM SP600125 at 24 hours. Scale bars = 300 μm. (C) Graph indicates the migration ability of stem cells into the wound area. Data are shown as the mean ± SD (n = 3, *P<0.05, ***P<0.005 versus control cells, ###p<0.005 versus IL-1β treated cells).

To investigate whether JNK, p38 MAPK and Akt signaling are also involved in IL-1β induced MMP-3 expression on hUCMSCs, hUCMSCs were pretreated with 25 nM p38 MAPK inhibitor SB203580, 20 μM Akt inhibitor GSK690693 and 20 nM JNK inhibitor SP600125 for 2 hours and then simulated with IL-1β for 36 hours. Q-PCR data showed that IL-1β induced MMP-3 mRNA can be inhibited by all these inhibitors (Fig 6A). To clarify the effects of these inhibitors in MMP3 expression, hUCMSCs were treated with ERK1/2 inhibitor U0126 (20 μM), p38 inhibitor SB205380 (50 nM), Akt inhibitor GSK690693 (20 μM), and JNK inhibitor SP600125 (20 nM) for 2 hours then incubated with or without IL-1β (100 ng/ml) in the continued presence of these inhibitors for 36 hours. The MMP-3 expression from the lysates of hUCMSCs after treatment with inhibitors followed by incubation without/with IL-1β were detected by Western blot. The results showed that without IL-1β stimulation, the MMP3 expression was not affected by these inhibitors. However, IL-1β-induced MMP-3 protein expression was suppressed by these inhibitors (S1 Fig). To determine the role of JNK, p38 MAPK and Akt signaling pathways in IL-1β-induced cell migration, wound healing assays were performed. As shown in Fig 6B and 6C, the migration ability of IL-1β-induced hUCMSCs was attenuated when JNK, p38 MAPK and Akt inhibitors were added prior to IL-1β stimulation. These results demonstrate that IL-1β- induced MMP-3 protein expression on hUCMSCs is mediated via JNK, p38 MAPK and Akt signaling pathways.

## Discussion

Recently, Bayo et al. indicated that autocrine motility factor (AMF) produced by hepatocellular carcinoma (HCC) was found to induce migration of different sources of MSCs *in vitro* and that exogenous stimulation of MSCs with recombinant AMF (rAMF) also promote the MSCs adhesion ability to endothelial cells due to the changes in the expression levels of MMP-3 [23]. AMF-primed MSCs increased the *in vivo* migration of MSCs towards experimental HCC tumors. In the present study, we found that IL-1β induced MMP-3 expression in hUCMSCs and enhance cell migration.

It has been found that IL-1β can stimulate lymphocyte and eosinophil cell migration [24, 25]. In this study, we found that pro-inflammation cytokine IL-1β enhances mesenchymal stem cell migration but does not influence cell proliferation. The gene expression profile between IL-1β treated and untreated control cells were identified by cDNA microarray, the results show that MMP-3 was up-regulated significantly in IL-1β treated mesenchymal stem cells. The level of MMP-3 transcripts was higher in the IL-1β treated stem cells when compared with non-treated cells using real-time PCR. The mRNA expression of MMP-3 was further supported by Western blotting analysis and ELISA assay: stem cells treated with IL-1β were shown to promote the expression of MMP-3 and secretion of substantial amounts of MMP-3 into the culture supernatants. MMP-3 is a kind of matrix metalloproteinase which degrades collagen types III, IV, and V, and it has been reported that MMP-3 could promote the invasive ability of human T4-2 cells and efficiently chemoattract embryonic cells [21]. It seems that MMP-3 plays an important role in extracellular matrix degradation and further effects on cell migration. In our study, MMP-3 activity and protein expression were suppressed by MMP-3 inhibitors ALX 260165 and UK 356618. Moreover, the presence of ALX 260165, UK 356618 or MMP-3 knockdown blocked IL-1β-induced MSCs migration and invasion. These results suggested that IL-1β-induced MMP-3 expression was response to the hUCMSCs migration.

IL-1β-mediated MMP-3 expression has be found in bone marrow MSCs [9], trabecular meshwork [19], and chondrocytes [20], possibly through activation of ERK1/2 cascade [19, 20]. Whether IL-1β-induced MMP-3 expression of stem cells occurs through MAPK Family and AKT signaling pathways were examined in this study. We found that IL-1β-induced MMP-3 mRNA, protein expression and MMP-3 activity were significantly decreased by MAPK and AKT signaling pathways inhibitors. These inhibitors also reduce the IL-1β mediated migration and invasion ability in hUCMSCs. Thus, it appears that IL-1β promotes the level of MMP-3 expression through MAPK and Akt signaling pathways to increase the migration and invasion ability of stem cells.

Human mesenchymal stem cells have been observed homing to and localizing in bone marrow, spleen, and mesenchymal tissues after intravenous infusion into unconditioned adult nude mice [26]. Stromal cell-derived factor (SDF)-1α [27], transforming growth factor (TGF)-β1, monocyte chemotactic protein (MCP)-1, and tumor necrosis factor (TNF)-α [2, 7] have been found to affect MSCs trafficking to injured regions.

Stem cell homing may provide an important clinical application of stem cells as treatment for various diseases. The potential therapeutic effect of MSCs depends on their ability to respond to migratory stimuli, break through physiological barriers blocking stem cell migration and engraft into a target tissue. Strategies for enhancing MSCs conditions may improve MSCs homing ability to injured tissues [2]. In our present study, we found that IL-1β stimulation enhanced hUCMSCs migration ability. Moreover, the expression of stemness of hUCMSCs after IL-1*β* treatment exhibited no significant difference in comparison with untreated hUCMSCs (S3 Fig), suggested that IL-1*β* treated hUCMSCs still have differentiation ability. Therefore, stimulate hUCMSCs with IL-1*β* could be a good strategy to enhance MSCs migrate to injured tissues.

## Conclusion

**Fig 7:**
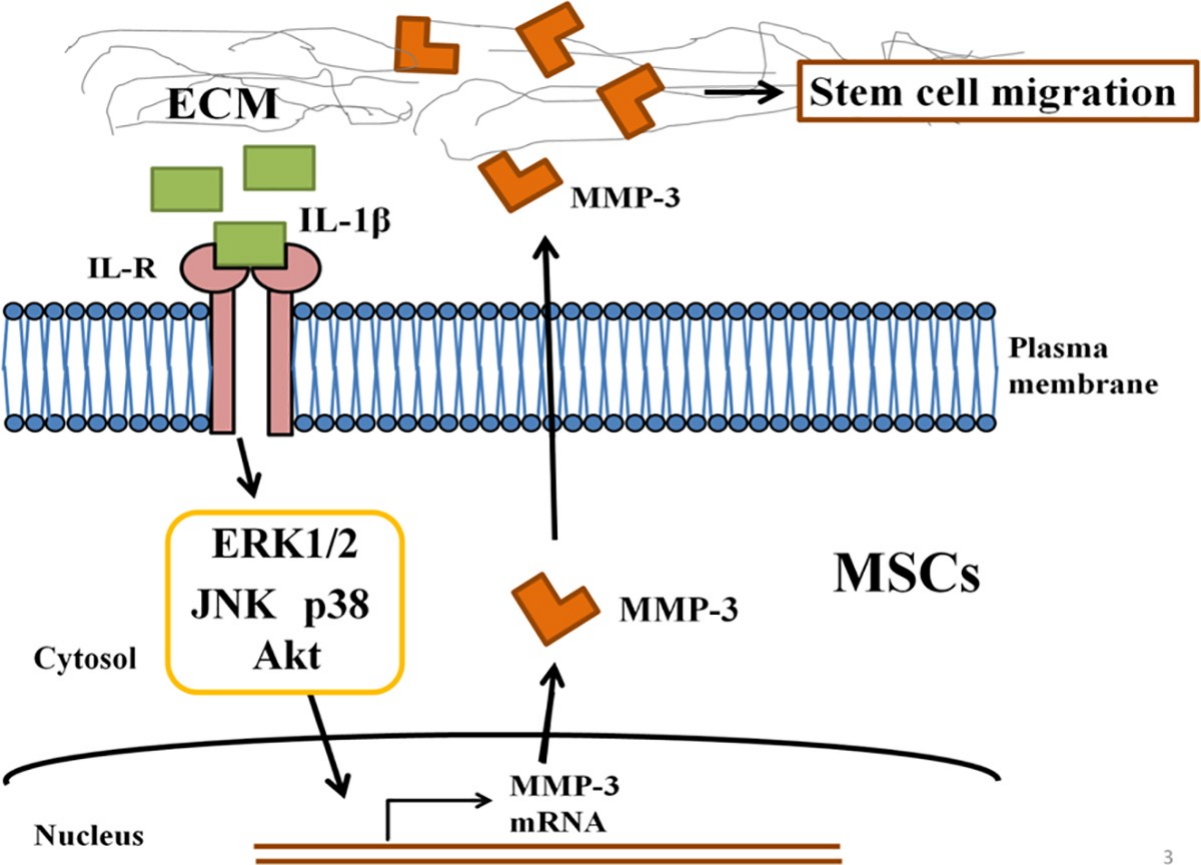
Schematic diagram of IL-1β signaling pathway in hUCMSCs migration. A schematic diagram depicts the proposed role of IL-1β signaling pathway in hUCMSCs migration. The process of cell migration is initiated by IL-1β through ERK1/2 induced expression of MMP-3 in hUCMSCs.

In conclusion, the results of this study suggest that the IL-1β induced MMP-3 expression is associated with stem cell migration, and that MAPK and AKT signaling pathways are involved in IL-1β mediated MMP-3 expression in promoting mesenchymal stem cell migration (Fig 7).

## Supporting information

S1 Fig Effects of ERK1/2, JNK, p38, and Akt pathways on IL-1β induced MMP-3 expression on hUCMSCs. hUCMSCs were treated with ERK1/2 inhibitor U0126 (20 μM), p38 inhibitor SB205380 (50 nM), Akt inhibitor GSK690693 (20 μM), and JNK inhibitor SP600125 (20 nM) for 2 hours then incubated with or without IL-1β (100 ng/ml) in the continued presence of these inhibitors for 36 hours. The MMP-3 expression from the lysates of hUCMSCs after treatment with inhibitors following incubation without/with IL-1β were detected by Western blot. The full-length Western blots were shown in S2 Fig.
(TIF)

S2 Fig The full-length Western blots. (TIF)

S3 Fig The expression of stemness markers on hUCMSCs. hUCMSCs were either left untreated or were treated with 100 ng/ml IL-1β for 36 hours. Control and treated cells were harvested, stained with stemness markers (CD105+, CD73+, CD90+, CD34−, CD45−), then analyzed using flow cytometry. The 1*10^4^ cells were collected in separate experiments. In each graph, the gray line was the unstained hUCMSCs; the black line was the unstained 100 ng/ml IL-1β treated hUCMSCs; the green line was the stained hUCMSCs; the red line was the stained 100 ng/ml IL-1β treated hUCMSCs.
(TIF)

## References

[1] Weiss ML, Medicetty S, Bledsoe AR, Rachakatla RS, Choi M, Merchav S, et al. Human umbilical cord matrix stem cells: preliminary characterization and effect of transplantation in a rodent model of Parkinson’s disease. Stem cells. 2006;24(3):781–92. 10.1634/stemcells.2005-0330 16223852

[2] Kang SK, Shin IS, Ko MS, Jo JY, Ra JC. Journey of mesenchymal stem cells for homing: strategies to enhance efficacy and safety of stem cell therapy. Stem cells international. 2012;2012. 10.1155/2012/342968 22754575 PMC3382267

[3] Gurtner GC, Werner S, Barrandon Y, Longaker MT. Wound repair and regeneration. Nature. 2008;453(7193):314–21. 10.1038/nature07039 18480812

[4] Li F, Huang Q, Chen J, Peng Y, Roop DR, Bedford JS, et al. Apoptotic cells activate the “phoenix rising” pathway to promote wound healing and tissue regeneration. Science signaling. 2010;3(110):ra13–ra. 10.1126/scisignal.2000634 20179271 PMC2905599

[5] Ponte AL, Marais E, Gallay N, Langonné A, Delorme B, Hérault O, et al. The in vitro migration capacity of human bone marrow mesenchymal stem cells: comparison of chemokine and growth factor chemotactic activities. Stem cells. 2007;25(7):1737–45. 10.1634/stemcells.2007-0054 17395768

[6] Askari AT, Unzek S, Popovic ZB, Goldman CK, Forudi F, Kiedrowski M, et al. Effect of stromal-cell-derived factor 1 on stem-cell homing and tissue regeneration in ischaemic cardiomyopathy. The Lancet. 2003;362(9385):697–703. 10.1016/S0140-6736(03)14232-8 12957092

[7] Fu X, Han B, Cai S, Lei Y, Sun T, Sheng Z. Migration of bone marrow‐derived mesenchymal stem cells induced by tumor necrosis factor‐α and its possible role in wound healing. Wound repair and regeneration. 2009;17(2):185–91. 10.1111/j.1524-475X.2009.00454.x 19320886

[8] Kollar K, Cook MM, Atkinson K, Brooke G. Molecular mechanisms involved in mesenchymal stem cell migration to the site of acute myocardial infarction. International journal of cell biology. 2009;2009. 10.1155/2009/904682 20130773 PMC2809335

[9] Carrero R, Cerrada I, Lledó E, Dopazo J, García-García F, Rubio M-P, et al. IL1β induces mesenchymal stem cells migration and leucocyte chemotaxis through NF-κB. Stem Cell Reviews and Reports. 2012;8(3):905–16. 10.1007/s12015-012-9364-9 22467443 PMC3412085

[10] Cheng CY, Hsieh HL, Sun CC, Lin CC, Luo SF, Yang CM. IL‐1β induces urokinse‐plasminogen activator expression and cell migration through PKCα, JNK1/2, and NF‐κB in A549 cells. Journal of cellular physiology. 2009;219(1):183–93. 10.1002/jcp.21669 19097143

[11] Lin C-C, Kuo C-T, Cheng C-Y, Wu C-Y, Lee C-W, Hsieh H-L, et al. IL-1β promotes A549 cell migration via MAPKs/AP-1-and NF-κB-dependent matrix metalloproteinase-9 expression. Cellular signalling. 2009;21(11):1652–62. 10.1016/j.cellsig.2009.07.002 19616091

[12] Mountain DJ, Singh M, Menon B, Singh K. Interleukin-1β increases expression and activity of matrix metalloproteinase-2 in cardiac microvascular endothelial cells: role of PKCα/β1 and MAPKs. American Journal of Physiology-Cell Physiology. 2007;292(2):C867–C75. 10.1152/ajpcell.00161.2006 16987994

[13] Tseng H-C, Lee I-T, Lin C-C, Chi P-L, Cheng S-E, Shih R-H, et al. IL-1β promotes corneal epithelial cell migration by increasing MMP-9 expression through NF-κB-and AP-1-dependent pathways. PLoS one. 2013;8(3):e57955. 10.1371/journal.pone.0057955 23505448 PMC3591450

[14] Wang F-m, Liu H-q, Liu S-r, Tang S-p, Yang L, Feng G-s. SHP-2 promoting migration and metastasis of MCF-7 with loss of E-cadherin, dephosphorylation of FAK and secretion of MMP-9 induced by IL-1 βin vivo andin vitro. Breast cancer research and treatment. 2005;89(1):5–14. 10.1007/s10549-004-1002-z 15666191

[15] Lin T, Wang X-l, Cai Y, Zettervall SL, Guzman RJ. Matrix Metalloproteinase-3 Regulates Arterial Calcification Arteriosclerosis, Thrombosis, and Vascular Biology. 2016;36(suppl_1):A370–A.

[16] Liu G, Bian S, Li F, Li X, Fan K, An H, et al. Effect of allogenic mesenchymal stem cells transplantation on the expression of interleukin-22 and matrix metalloproteinase-3 in rats with collagen induced arthritis. Zhonghua yi xue za zhi. 2017;97(9):698–702. 10.3760/cma.j.issn.0376-2491.2017.09.014 28297833

[17] Aftab Q, Mesnil M, Ojefua E, Poole A, Noordenbos J, Strale P-O, et al. Cx43-associated secretome and interactome reveal synergistic mechanisms for glioma migration and MMP3 activation. Frontiers in Neuroscience. 2019;13:143. 10.3389/fnins.2019.00143 30941001 PMC6433981

[18] Ries C, Egea V, Karow M, Kolb H, Jochum M, Neth P. MMP-2, MT1-MMP, and TIMP-2 are essential for the invasive capacity of human mesenchymal stem cells: differential regulation by inflammatory cytokines. Blood. 2007;109(9):4055–63. 10.1182/blood-2006-10-051060 17197427

[19] Kelley MJ, Rose AY, Song K, Chen Y, Bradley JM, Rookhuizen D, et al. Synergism of TNF and IL-1 in the induction of matrix metalloproteinase-3 in trabecular meshwork. Investigative ophthalmology & visual science. 2007;48(6):2634–43. 10.1167/iovs.06-1445 17525194

[20] Wang X, Li F, Fan C, Wang C, Ruan H. Effects and relationship of ERK1 and ERK2 in interleukin-1β-induced alterations in MMP3, MMP13, type II collagen and aggrecan expression in human chondrocytes. International journal of molecular medicine. 2011;27(4):583–9. 10.3892/ijmm.2011.611 21305249

[21] Zhao M, Sachs PC, Wang X, Dumur CI, Idowu MO, Robila V, et al. Mesenchymal stem cells in mammary adipose tissue stimulate progression of breast cancer resembling the basal-type. Cancer biology & therapy. 2012;13(9):782–92. 10.4161/cbt.20561 22669576 PMC3399703

[22] Chen M-S, Lin C-Y, Chiu Y-H, Chen C-P, Tsai P-J, Wang H-S. IL-1β-induced matrix metalloprotease-1 promotes mesenchymal stem cell migration via PAR1 and G-protein-coupled signaling pathway. Stem cells international. 2018;2018. 10.1155/2018/3524759 30026761 PMC6031215

[23] Bayo J, Fiore E, Aquino JB, Malvicini M, Rizzo M, Peixoto E, et al. Increased migration of human mesenchymal stromal cells by autocrine motility factor (AMF) resulted in enhanced recruitment towards hepatocellular carcinoma. PloS one. 2014;9(4):e95171. 10.1371/journal.pone.0095171 24736611 PMC3988162

[24] Bacon K, Westwick J, Camp R. Potent and specific inhibition of IL-8-, IL-1α-and IL-1β-inducedin vitro human lymphocyte migration by calcium channel antagonists. Biochemical and Biophysical Research Communications. 1989;165(1):349–54. 10.1016/0006-291x(89)91076-0 2686646

[25] Ebisawa M, Bochner BS, Georas SN, Schleimer RP. Eosinophil transendothelial migration induced by cytokines. I. Role of endothelial and eosinophil adhesion molecules in IL-1 beta-induced transendothelial migration. The Journal of Immunology. 1992;149(12):4021–8. 1460288

[26] Allers C, Sierralta WD, Neubauer S, Rivera F, Minguell JJ, Conget PA. Dynamic of distribution of human bone marrow-derived mesenchymal stem cells after transplantation into adult unconditioned mice. Transplantation. 2004;78(4):503–8. 10.1097/01.tp.0000128334.93343.b3 15446307

[27] Imitola J, Raddassi K, Park KI, Mueller F-J, Nieto M, Teng YD, et al. Directed migration of neural stem cells to sites of CNS injury by the stromal cell-derived factor 1α/CXC chemokine receptor 4 pathway. Proceedings of the National Academy of Sciences. 2004;101(52):18117–22. 10.1073/pnas.0408258102 15608062 PMC536055

