# Supplementary figures and images for: Interleukin-1β-induced matrix metalloproteinase-3 via ERK1/2 pathway to promote mesenchymal stem cell migration
Source: PLoS One. 2021 May 21;16(5):e0252163. doi: 10.1371/journal.pone.0252163 (PMC8139494; doi:10.1371/journal.pone.0252163)

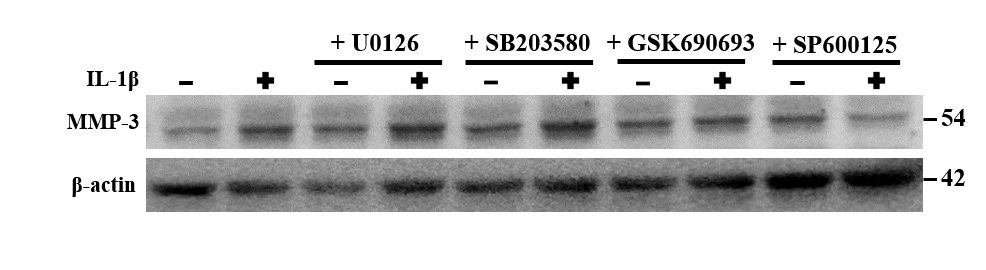

Supplement: S1 Fig — hUCMSCs were treated with ERK1/2 inhibitor U0126 (20 μM), p38 inhibitor SB205380 (50 nM), Akt inhibitor GSK690693 (20 μM), and JNK inhibitor SP600125 (20 nM) for 2 hours then incubated with or without IL-1β (100 ng/ml) in the continued presence of these inhibitors for 36 hours. The MMP-3 expression from the lysates of hUCMSCs after treatment with inhibitors following incubation without/with IL-1β were detected by Western blot. The full-length Western blots were shown in S2 Fig. (TIF) [file pone.0252163.s001.tif]

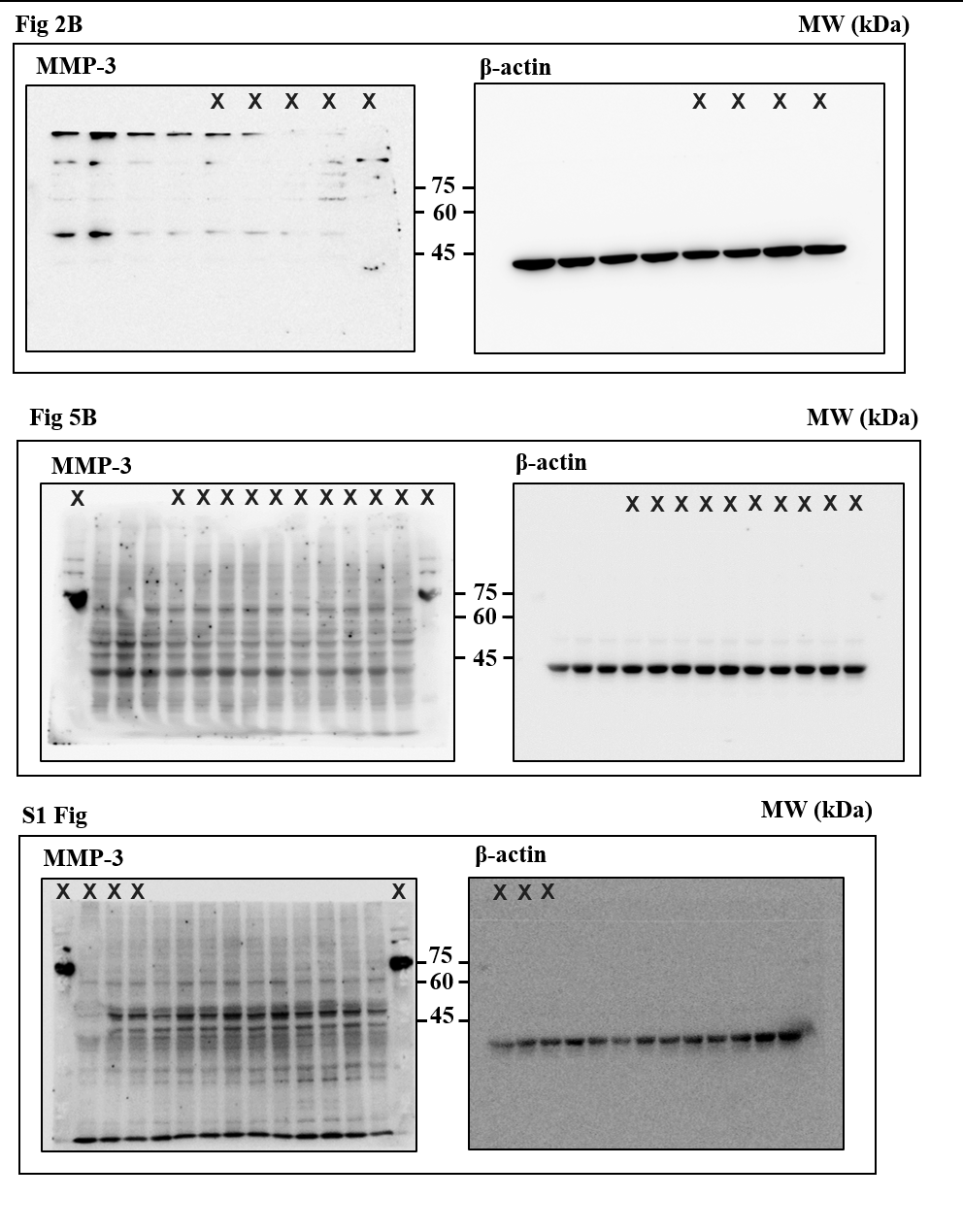

Supplement: S2 Fig — (TIF) [file pone.0252163.s002.tif]

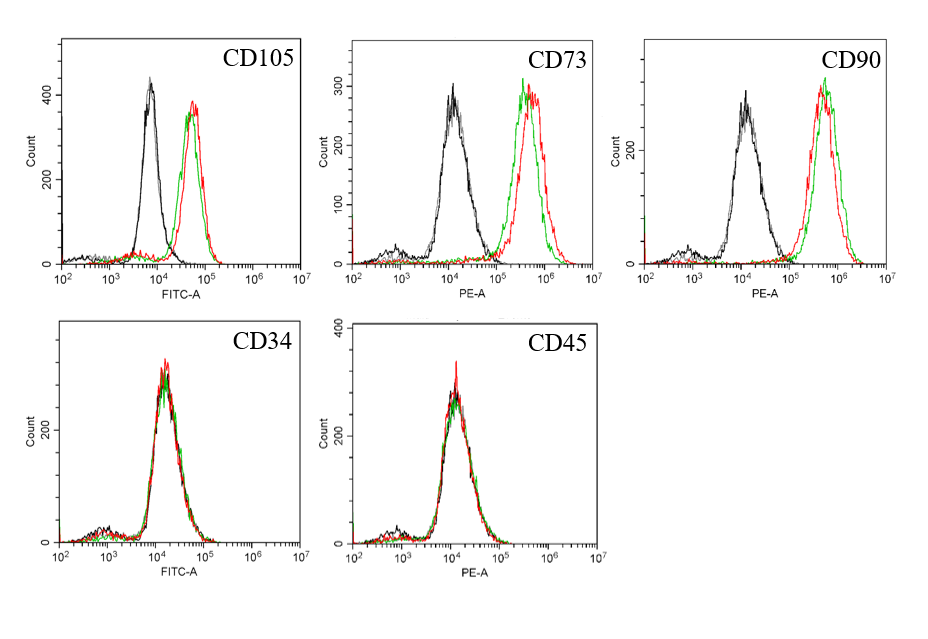

Supplement: S3 Fig — hUCMSCs were either left untreated or were treated with 100 ng/ml IL-1β for 36 hours. Control and treated cells were harvested, stained with stemness markers (CD105+, CD73+, CD90+, CD34−, CD45−), then analyzed using flow cytometry. The 1*104 cells were collected in separate experiments. In each graph, the gray line was the unstained hUCMSCs; the black line was the unstained 100 ng/ml IL-1β treated hUCMSCs; the green line was the stained hUCMSCs; the red line was the stained 100 ng/ml IL-1β treated hUCMSCs. (TIF) [file pone.0252163.s003.tif]
